# Wireless skin sensors for electrocardiogram and heart rate monitoring in the neonatal intensive care unit: a prospective feasibility, safety, and accuracy study

**DOI:** 10.3389/fbioe.2025.1555882

**Published:** 2025-04-29

**Authors:** Eva Senechal, Daniel Radeschi, Shasha Lv, Emilie Jeanne, Ana Saveedra Ruiz, Lydia Tao, Brittany Dulmage, Wissam Shalish, Robert Edward Kearney, Guilherme Sant’Anna

**Affiliations:** ^1^ Experimental Medicine Department, McGill University Health Center, Montreal, QC, Canada; ^2^ Biomedical Engineering Department, McGill University, Montreal, QC, Canada; ^3^ Research Institute of the McGill University Health Center, Montreal, QC, Canada; ^4^ Department of Dermatology, Ohio State University Wexner Medical Center, Columbus, OH, United States; ^5^ Division of Neonatology, Department of Pediatrics, McGill University Health Center, Montreal, QC, Canada; ^6^ Biomedical Engineering Department, McGill University, Montreal, QC, Canada

**Keywords:** NICU (neonatal intensive care unit), wireless technologies, wireless sensor, heart rate, patient monitoring, bedside monitoring, electrocadiography

## Abstract

**Objectives:**

Assess feasibility, safety, and accuracy of electrocardiogram (ECG) and heart rate (HR) monitoring in neonates, using a new wireless skin sensor.

**Methods:**

Prospective observational study in infants of any gestational age admitted in the neonatal intensive care unit. ECG/HR signals were simultaneously recorded from a standard wired and new wireless system with bedside annotations. Feasibility was evaluated as signal coverage, gap numbers/durations, and sources of gaps. Safety was appraised by changes in skin condition and pain after/upon wireless sensor removal. Accuracy was measured using bias and 95% limits of agreement, and the coefficient of determination. The ability of the wireless sensors to detect normal and abnormal HR values was evaluated using a Clark Error Grid. Additionally, user satisfaction from parents and nurses were appraised using a short questionnaire.

**Results:**

25 infants had 757 h of recorded signals over 96 days. ECG coverage was 99.9% [IQR: 99.9%–99.95%] for the wired vs 97.8% [IQR: 81.6%–99.9%; p < 0.00] for the wireless system, while HR coverage was 99.4% [IQR: 98.6%–99.9%] vs 89.7% [IQR: 75.6%–97.6%; p < 0.00]. Wireless ECG gaps were <5 s in 97% of cases, and HR gaps <30 s in 85%. All ECG gaps and 57% of HR gaps were due to Bluetooth disconnection (BD). 78% of BD in wireless HR were during kangaroo care (78%). Of 192 skin photographs (96 pairs), 98% were taken, showing increased but low skin scores post-removal, with median pain scores also low. Accuracy metrics showed strong agreement, with the Clark Error Grid indicating 97% of paired signals led to the same clinical outcome. Among 23 nurse and 18 parent responses, satisfaction with the wireless system was high.

**Conclusion:**

ECG and HR monitoring using a new wireless skin sensor was feasible, safe, and accurate when compared to the wired standard. Future adjustments in the technology are needed to improve signal coverage during handling and KC and test the sensors in unstable and more immature patients. Limitations included challenges in recruiting unstable neonates, variability introduced by multiple raters completing pain assessments, and inability to apply safety metrics to the wired standard of care.

## 1 Introduction

In neonatal intensive care, continuous monitoring of vital signals such as ECG and heart rate (HR) is a standard practice (Kumar et al., 2020). This is usually attained by using three skin sensors connected to a large bedside monitor via wires and cables. Unfortunately, as the patient moves the wires may tangle around the body and cause discomfort, pain, pressure sores, and/or limit optimal patient positioning (Bonner et al., 2017). Additionally, wires may break, disconnect, or become soiled by body secretions or touch the floor during Kangaroo Care (KC), requiring frequent replacement or cleaning (Russotto et al., 2015; Bonner et al., 2017). Furthermore, the presence of wires and cables usually leads to the perception of a highly technical environment making parents feel intimidated and nervous to touch their baby or engage in KC (Al Maghaireh et al., 2016; Conde-Agudelo and Díaz-Rossello, 2016; Bonner et al., 2017; Mehrpisheh et al., 2022). Therefore, although current wired monitoring system have advanced tremendously patient care, it can increase nursing workload and lead to neonatal distress and parental anxiety (Bonner et al., 2017).

Interestingly, in what concerns monitoring connectivity, vital signals technologies in the NICU have not seen major changes over the last 3 decades (Xu et al., 2021). In part, this gradual progress is related to the challenges for conducting pivotal trials in neonatal acute care settings (De Georgia et al., 2015; Christodoulakis et al., 2017; Sertkaya et al., 2022). Any new monitoring system needs to be feasible, safe, and accurate for use in a complex hospital environment.

## 2 Literature review

There has been a growing interest in the development of wireless technologies for hospitalized pediatric patients, with a particular focus on NICU patients (Senechal et al., 2023a; Krbec et al., 2024). However many of these studies have used short monitoring durations in a small sample size under well controlled experimental conditions (Chung et al., 2019; Tanigasalam et al., 2019; Chen et al., 2020; 2024; Grooby et al., 2021; Henry et al., 2021; Kwak et al., 2021; Pike et al., 2021; Sriraam et al., 2021; Harrell et al., 2022; Rao et al., 2023; Senechal et al., 2023a; Yoo et al., 2023; Thomas et al., 2024). Many studies focused on older, more stable infants, excluding those born prematurely or requiring interventions like respiratory support (Coleman et al., 2022; Ginsburg et al., 2022b; Harrell et al., 2022). Furthermore, studies often excluded periods of poor signal quality, data collected during patient care activities, or omitted data from analysis without providing a clear justification (Chung et al., 2020; Pike et al., 2021; Harrell et al., 2022; Thomas et al., 2024).

In a proof-of-concept study in NICU patients, a novel wireless monitoring system consisting of two small skin sensors was tested and evaluated in only six patients (Chung et al., 2020). Two other studies examined this system and concluded that measurements of HR were similar to the pulse rate values obtained with an oximeter (Rad 97 Massimo Corporation, United States) (Coleman et al., 2022; Ginsburg et al., 2022b). However, these studies did not include NICU patients, used large HR averaging windows, and inconsistent recording periods. Thus, a more comprehensive and longer evaluation under realistic NICU conditions is necessary to advance the field.

## 3 Objective

The objective of this study was to investigate the feasibility, safety, and accuracy of ECG and HR monitoring using a new wireless system during routine NICU care.

## 4 Methods

### 4.1 Study design

A single center, prospective, observational study was conducted at the Montreal Children’s Hospital NICU from August 2022 to March 2023. The study protocol was registered in clinicaltrials.gov [NCT04956354] and published (Senechal et al., 2023b). The protocol was approved by the Research Ethics Board of the McGill University Health Center (#2022-7704). Written informed consent was obtained from parents of all participating infants before enrollment.

### 4.2 Participants

Infants admitted in the NICU of any gestational age (GA) and with or without any type of respiratory support were eligible in an effort to recruit a diverse and representative set of patients. Infants with congenital skin infections or fragile skin, or major congenital anomalies were excluded.

### 4.3 Study equipment and recording

**FIGURE 1:**
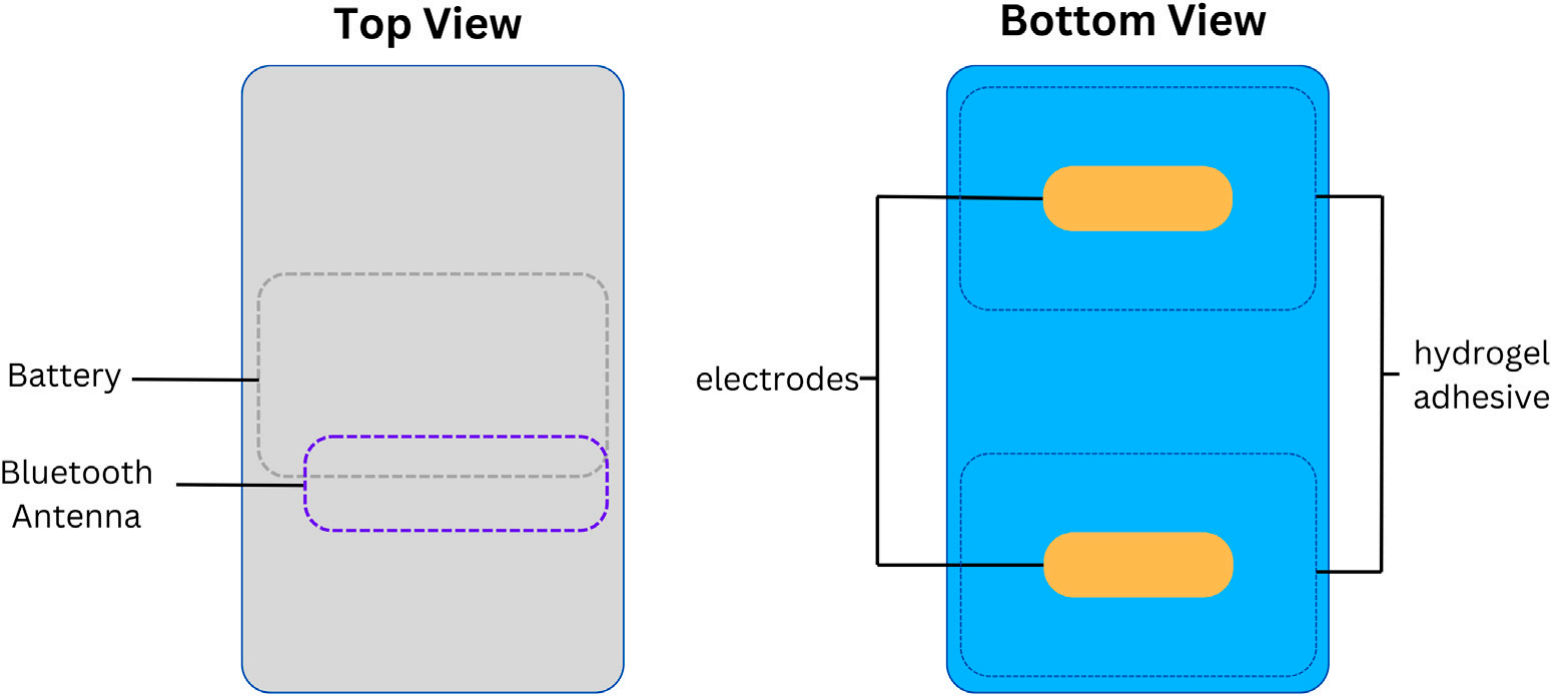
Wireless skin sensor components. Legend. Design and internal components of the wireless chest senor unit.

**FIGURE 2:**
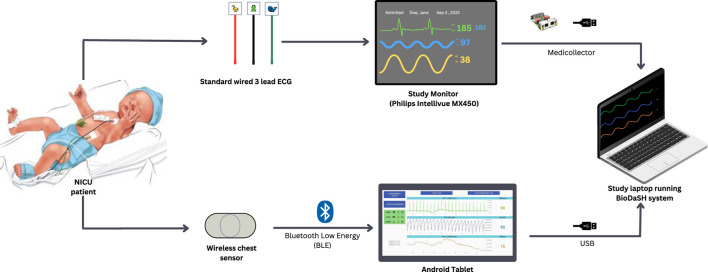
Electrocardiography and heart rate recordings (study design). Legend. NICU: neonatal intensive care unit; ECG, electrocardiography; USB, Universal Serial Bus. Simultaneous recordings of both systems were possible using Biosensor Data Aggregation and Synchronization (BioDaSH) system.

In the NICU, infants were monitored with the standard wired system (Philips Intellivue MX450, Philips, Netherlands) using the Neotrode ECG leads (Neotrode, ConMed, United States). For the study, these wires were disconnected from the beside monitor and connected to a dedicated monitor used exclusively for the study (Phillips Intellivue MX 450). A wireless skin sensor (ANNE^®^ One, Sibel Health Inc., United States) was placed on the chest using a one-time hydrogel adhesive. The chest unit included two ECG electrodes and a Bluetooth Low Energy System (Figure 1). Wireless signals were transmitted to a research Android tablet running a custom data recording application specifically designed for the study. Then, the wired monitor and the tablet transmitted the signals to a research laptop where a Biosensors Data Aggregation and Synchronization (BioDASh) application simultaneously acquired data from the Philips monitor via MediCollector (MediCollector, United States) and from the tablet via USB connection (Figure 2). BioDASh also enabled live text annotations during recordings. All recorded data was securely transferred to Dropbox and stored on a hard drive using the *Parquet* file format. Each patient was monitored for 8 hours a day over four consecutive days during routine NICU care. Study investigators remained at the bedside during this period for annotations, to ensure quality of signals recordings, and to ensure that the study would not interfere with patient care. Patient demographics were gathered using a data collection form including date of birth, age, GA, postmenstrual age (PMA) at time of the study, birth weight, weight at time of the study, and clinical diagnoses (see Supplementary Material S1).

### 4.4 Primary objective

The primary objective was to assess for feasibility, safety, and accuracy of monitoring ECG and HR using a new wireless system when compared to a standard wired system, during routine NICU care.

### 4.5 Signal processing

Prior to analysis, ECG and HR signals were preprocessed using a custom algorithm that resampled the signals to a common and uniform rate (250 Hz for ECG and 1 Hz for HR) and synchronized corresponding signals (Radeschi et al., 2023). All data was processed and analyzed using MATLAB R2023b (MathWorks, United States).

### 4.6 Data analysis

Feasibility was evaluated by assessing ECG and HR signal *coverage* and *gaps*. *Coverage* was defined as the percentage of recording duration for which the signal was available, and *Gap* was defined as any period with no signal value available for >8 m for ECG and >2s for HR. Then, the distribution of gap lengths for the wired and wireless systems were calculated. After that, to identify the *source of gaps* three unique sets of variables were assessed: (1) alert signals automatically generated by the wireless sensor for Bluetooth connectivity and ECG lead skin contact; (2) annotations manually recorded by the research team (see Supplementary Material S2 for full list), and (3) signal-to-noise ratio (SNR) continuously estimated from the underlying ECG. Thus, gaps were categorized as the result of one of four mechanisms: (1) Bluetooth disconnections (BD), (2) sensor/lead removals or adjustments, (3) low SNR in the ECG (defined as SNR <5 dB (Smital et al., 2020), or (4) unknown causes. The third mechanism (low SNR) is illustrated in Figure 4A through 4E.

Safety was only assessed for the new wireless sensor as the standard wired sensors were already on the infant skin before the start of the recordings and remained in place after the end. Safety assessment consisted of evaluating: (1) *changes in skin condition* by comparing skin photos taken with an 8-megapixel camera resolution of an iPad (9^th^ generation, Apple) before placement and after removal of the wireless sensor. Photos were de-identified and a Neonatal Skin Condition Score (NSCS), a widely used and validated metric of skin condition, was determined by a board-certified dermatologist blinded to the study; NSCS scores range from 3 (intact) to 9 (very poor); (2) *indications of pain* during sensors removal by applying the Neonatal Infant Pain Scale (NIPS); NIPS score range from 0 (painless) to 7 (very painful) with scores of 3 or greater considered to be indicative of at least some pain (Lund and Osborne, 2004; Rana et al., 2017; Sarkaria and Gruszfeld, 2022).

Accuracy was assessed by applying several statistical measures to corresponding wired and wireless HR signals on a sample-by-sample basis. These measures included the *bias* and *95% limits of agreement*, derived from a Bland-Altman analysis, and the *coefficient of determination* ( $R^{2}$ ) (Martin Bland and Altman, 1986). The ability of the wireless sensors to detect bradycardia (HR < 100bpm) and tachycardia (HR > 180bpm) was assessed as a secondary accuracy outcome. Furthermore, the clinical implications of differences between the wired and wireless systems were assessed using a modified Clark Error Grid Analysis. Region descriptions and modifications from the original EGA are specified in the Supplementary Material S3 (Clarke et al., 1987).

Additionally, as a secondary outcome, we evaluated user satisfaction in parents and nurses of infants that participated in the study. The questionnaire can be found in supplementary documents (Supplementary Material S4).

### 4.7 Sample size and statistical analysis

A convenience sample of 24 neonates providing 96 recording sessions (768 h) was chosen due to lack of *a priori* knowledge regarding variance in the wireless ECG and HR signals. In the feasibility assessment, signal coverage for wired and wireless ECG and HR was computed for each day of recording; corresponding distributions of wired and wireless coverages were then compared using a Wilcoxon signed-rank test with a significance level of 0.1%. Distributions of gaps lengths were created for each system and compared. The source of gaps was evaluated by the percentage of cumulative recording time with no signal available and coinciding with each mechanism. Finally, cumulative SNR probability distributions were computed and presented for each system; distributions were compared using a Wilcoxon rank sum test with a significance level of 0.1%. Safety was assessed by using a Wilcoxon signed rank to assess for differences between NSCS before and following device removals. An additional Wilcoxon signed-rank test was conducted to evaluate if the NIPS scores obtained were significantly higher than the pain threshold of 3. Accuracy was assessed by statistical agreement metrics for each day of recording using the distribution and the median and interquartile range (IQR) for each metric. Further, effect sizes for each metric with a significance level of 5% and study power of 90% were calculated. In the error grid analysis, the percentage of sample pairs contained within each region of the grid was computed.

## 5 Results

**FIGURE 3:**
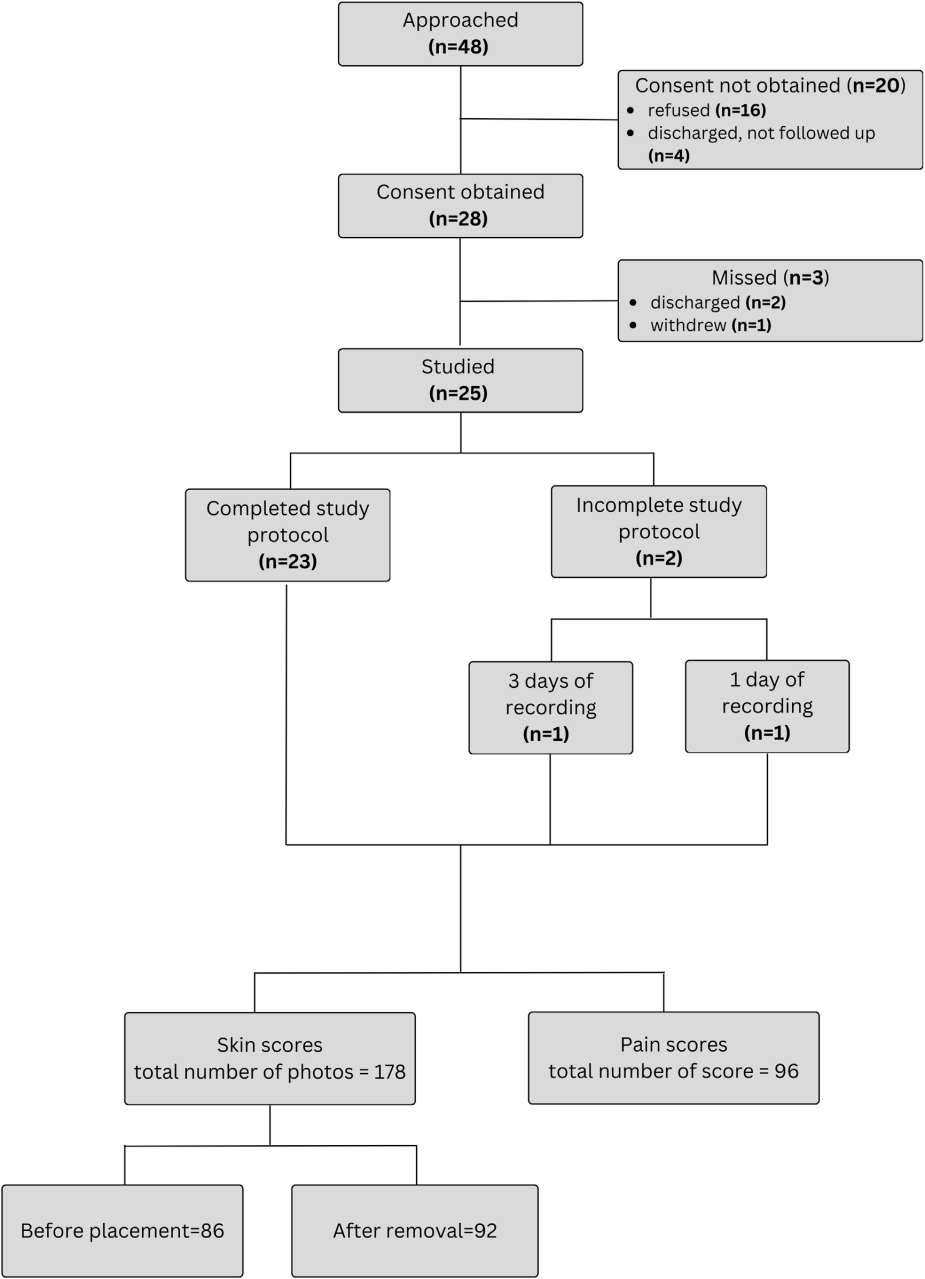
Recruitment and data collection flow chart.

Forty-eight patients were approached for the study. The flowchart of study enrollment is provided on Figure 3. Ultimately total of 96 days of recordings were performed on 25 infants. An additional participant was recruited to compensate for one patient discontinuing after only 1 day (8 h) and another that withdrew after 3 days (24 h) of recordings (Figure 3). One withdrawal was due to mild bruising below the chest sensor, and the other occurred at the parents’ request due to perceived discomfort and tachypnea during sensor removal. As some daily recordings were shorter than 8 h because of interruptions for exams and procedures requiring sensor removal, a total of 757 h (99%) of simultaneous recordings with both systems were obtained. Twenty-two of the included patients were preterm infants. During recordings, 13 infants (52%) were receiving nasal continuous positive airway pressure (CPAP), 3 (12%) on mechanical ventilation, and 9 (36%) were stable in room air. Patient characteristics are summarized in Table 1 and information about diagnoses at enrollment in Table 2.

**TABLE 1:** Clinical characteristics.

	All (n = 25	CPAP (n = 13)	Room air (n = 9)	CMV (n = 3)
Gestational Age (weeks)	28.4 [26.1–30.7] (25–40.7)	28.4 [26.6–29.3] (25.3–32)	30.7 [28.3–35.8] (26.1–40.7)	25.6 [25.1–26.0] (25–26.1)
Corrected gestational age (weeks)	33.3 [31.3–36.1] (26.3–45.9)	33.1 [31.2–33.8] (26.3–36.7)	36.3 [35.1–40.8] (32.1–45.9)	27.3 [27.0–29.1] (26.9–29.7)
Birthweight (g)	1,110 [780–1,397] (600–3,480)	1,200 [806–1,356] (600–1790)	1,360 [795–2095] (605–3,480)	780 [728–1,020] (710–1,100)
Current weight (g)	1,450 [1,151–1930] (750–3,990)	1,450 [1,225–1795] (810–2,685)	1870 [1,378–3,028] (1,150–3,990)	1,000 [813–1,030] (750–1,040)

Results are reported as median [IQR] and (min-max). Continuous positive airway pressure (CPAP), and conventional mechanical ventilation (CMV).

**TABLE 2:** Active diagnosis at enrollment.

Diagnosis	Infants studied N = 25
Apneas and Bradycardia events	10 (40)
Anemia	8 (32)
Hyperbilirubinemia	8 (32)
Respiratory Distress Syndrome	8 (32)
Intraventricular Hemorrhage	5 (20)
Lung Immaturity	4 (16)
Intrauterine Growth Restriction	2 (8)
Patent Ductus Arteriosus	2 (8)
Bronchopulmonary Dysplasia	1 (4)
Cholestasis	1 (4)
Feeding Intolerance	1 (4)
Gastric Perforation w/ileostomy	1 (4)
Gastro-Esophageal Reflux	1 (4)
Suspected Neonatal Sepsis	1 (4)
Urinary Tract Infection	1 (4)

Results are presented as n (%); some participants had more than one diagnosis.

### 5.1 Feasibility

#### 5.1.1 Coverage

**FIGURE 4:**
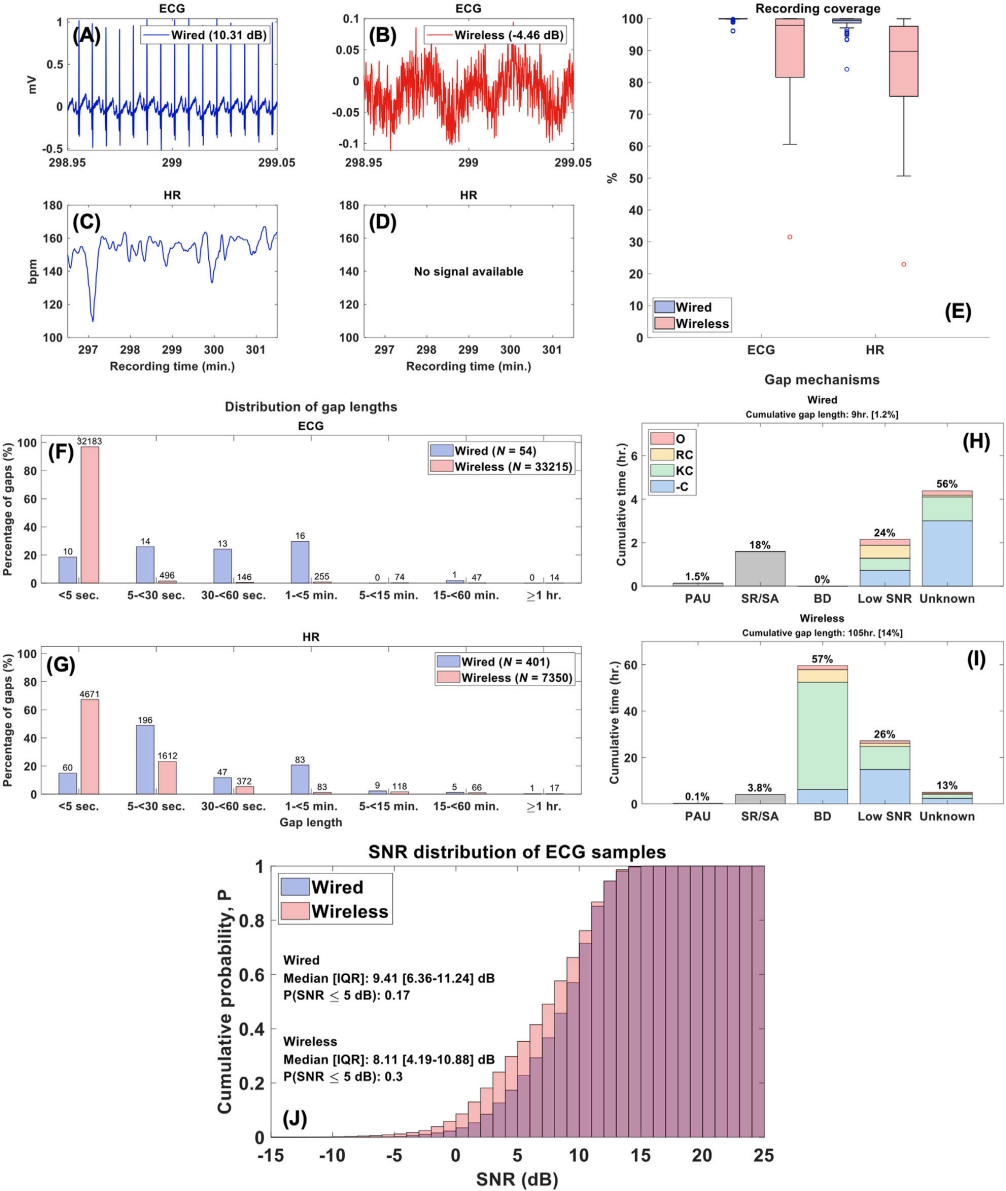
Feasibility Analysis. Legend. **(A–D)** ECG and HR segments from a typical patient record. The wired ECG had an estimated SNR of 10.31 dB and HR values were available, while the wireless had an SNR of -4.46 dB with no HR information. **(E)** Distribution of sample coverage for ECG and HR recordings. **(F, G)** Distributions of gap lengths across all wired and wireless ECG and HR recordings. The labels above each bar reflect the total gap count in the respective groups and system. **(H, I)** Correlation between gap mechanisms and periods of missing data in HR signals and the distribution of prominent annotations across these mechanisms. The cumulative gap length represents the total time with no signal available across 757 hours of recording [including % of recording time] and percentages above bars reflect % of cumulative gap lengths; [PAU, pause, SR/SA = sensor removed/adjusted, BD, Bluetooth disconnection, -C, no comment, KC, kangaroo care, RC, routine care, O, other]. **(J)** Cumulative probability distributions of SNR estimates across ECG samples.

The median [IQR] coverage of the wired ECG signal was 99.9% [IQR: 99.9%–99.95%] *versus* 97.8% of the wireless [IQR: 81.6%–99.9%] (*p*-value <0.001) and for HR values was 99.4% [IQR: 98.6%–99.9%] vs. 89.7% [IQR: 75.6%–97.6%] (*p*-value <0.001), for the wired and wireless systems, respectively (Figures 4A–E).

#### 5.1.2 Gaps

ECG and HR wireless signals contained more interruptions than the wired system: 33,215 vs. 54 and 7,350 vs. 401, respectively. Thus, of the 757 h of recording, the wireless signal had 105 h (14%) of missing data, while the wired HR signal had 9 h (1%). Importantly, 32,183 (97%) disruptions in the wireless ECG signals were very short (<5 s) and 6,283 (85%) interruptions of HR values were <30 s (Figures 4F, G).

#### 5.1.3 Source of gaps

In the wired system, ECG gaps were solely the result of lead disconnections; in the wireless system, these gaps were the result of BD. Figures 4H, I present the complete distribution of gap mechanisms across missing HR data. Most of the gaps for HR values (56%; 5 h) with the wired system were due to unknown mechanisms with some related to poor ECG signal quality with an SNR <5 dB (24%; 2.2 h). In contrast, the majority of HR gaps in the wireless system were linked to Bluetooth disconnections (57%; 59.9 h) occurring during manipulation, primarily for KC (78%) or routine nursing care (9%). The cumulative probability distributions of the SNR in the wired and wireless ECG signals are presented in Figure 4J. The median SNR across all wired ECG samples was 9.41 [IQR: 6.36–11.24] dB *versus* 8.11 [IQR: 4.19–10.88] dB in the wireless system (p-value <0.001).

### 5.2 Safety

**FIGURE 5:**
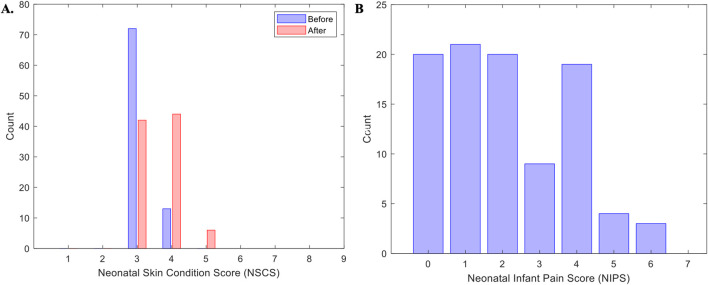
Skin and pain scores. Panel **(A)** represents the distribution of all patients score for the Neonatal Skin Condition Score (NSCS) across all days of recordings. Panel **(B)** represents the Neonatal infant pain Scale (NIPS) across all patients and all days of recordings.

A total of 188/192 (98%) skin photographs (96 pairs) were taken during the study; 4 (2%) were not taken before sensor placement. Furthermore, 10/188 (5%) photos were deemed of insufficient quality for scoring, leaving a total of 178 (93%) photos (86 pre-placement and 92 post-placement) available. Of the 86 pre-placement photos, all infants exhibited either a score of 3 (n = 73, 85%) or 4 (n = 13, 15%). Following removal of the device most infants skin scores remained low at 3 (n = 42, 47%) or 4 (n = 44, 50%), and four infants showed a score of 5 (n = 4, 4%). A Wilcoxon Signed Rank Test found statistically significant difference in NSCS between pre- and post-removal (z = −5.468, p < 0.001). A total of 82 (85%) recordings had before-and-after photos for comparative analysis: 40 (49%) pairs no change, 36 (44%) increase of one point, and 3 (4%) an increase of two points. Detailed skin scores and the distribution of differences before-and-after removal are presented (Table 3; Figure 5A). A total of 96 Neonatal Infant Pain Scale (NIPS) scores were obtained (Table 3; Figure 5B). The median NIPS scores was <3 (z = − 4.64, p = 1.68 $e^{-6}$ ) with a mean of 2.10 (±1.70, median = 2, IQR: 1–4). There was no correlation between skin photos scores and pain scores. Additional analysis on daily skin and pain scores is available as Supplemental Material (Supplementary Material S5).

**TABLE 3:** Daily skin and pain score distribution.

Neonatal Skin Condition Score (NSCS)									
Day	Time/Score	3	4		5		6–9		Total
Day 1	Before	18 (10)	4 (2)		-		-		22 (12)
	After	10 (6)	14 (8)		1 (0.6)		-		25 (14)
Day 2	Before	19 (10)	4 (2)		-		-		23 (13)
	After	12 (68)	9 (5)		2 (1)		-		23 (13)
Day 3	Before	18 (10)	2 (1)		-		-		20 (11)
	After	7 (4)	14 (8)		2 (1)		-		23 (13)
Day 4	Before	18 (10)	3 (2)		-		-		21 (12)
	After	13 (7)	7 (4)		1 (0.6)		-		21 (12)
	Total	115 (65)	57 (32)		6 (3)		-		178 (100)
∆ NSCS (after-before)									
	−1		0		1		2		Total
Day 1	0		12 (15)		11 (13)		0		23 (27)
Day 2	2 (3)		10 (12)		9 (11)		1 (1)		22 (27)
Day 3	0		6 (7)		11 (13)		1 (1)		18 (23)
Day 4	1 (1)		12 (15)		5 (6)		1 (1)		19 (23)
Total	3 (4)		40 (49)		36 (44)		3 (4)		82 (100)
Neonatal Infant Pain Scale									
	0	1	2	3	4	5	6	7	Total
Day 1	6 (6)	7 (7)	2 (2)	3 (3)	5 (5)	2 (2)	-	-	25 (26)
Day 2	5 (5)	5 (5)	6 (6)	2 (2)	4 (4)	1 (1)	1 (1)	-	24 (25)
Day 3	5 (5)	6 (6)	5 (5)	1 (1)	6 (6)	-	1 (1)	-	24 (24)
Day 4	4 (4)	3 (3)	7 (7)	3 (3)	4 (4)	1 (1)	1 (1)	-	23 (24)
Total	20 (21)	21 (22)	20 (21)	9 (9)	19 (20)	4 (4)	3 (3)	-	96 (100)

Results are presented as n (%).

### 5.3 Accuracy

**FIGURE 6:**
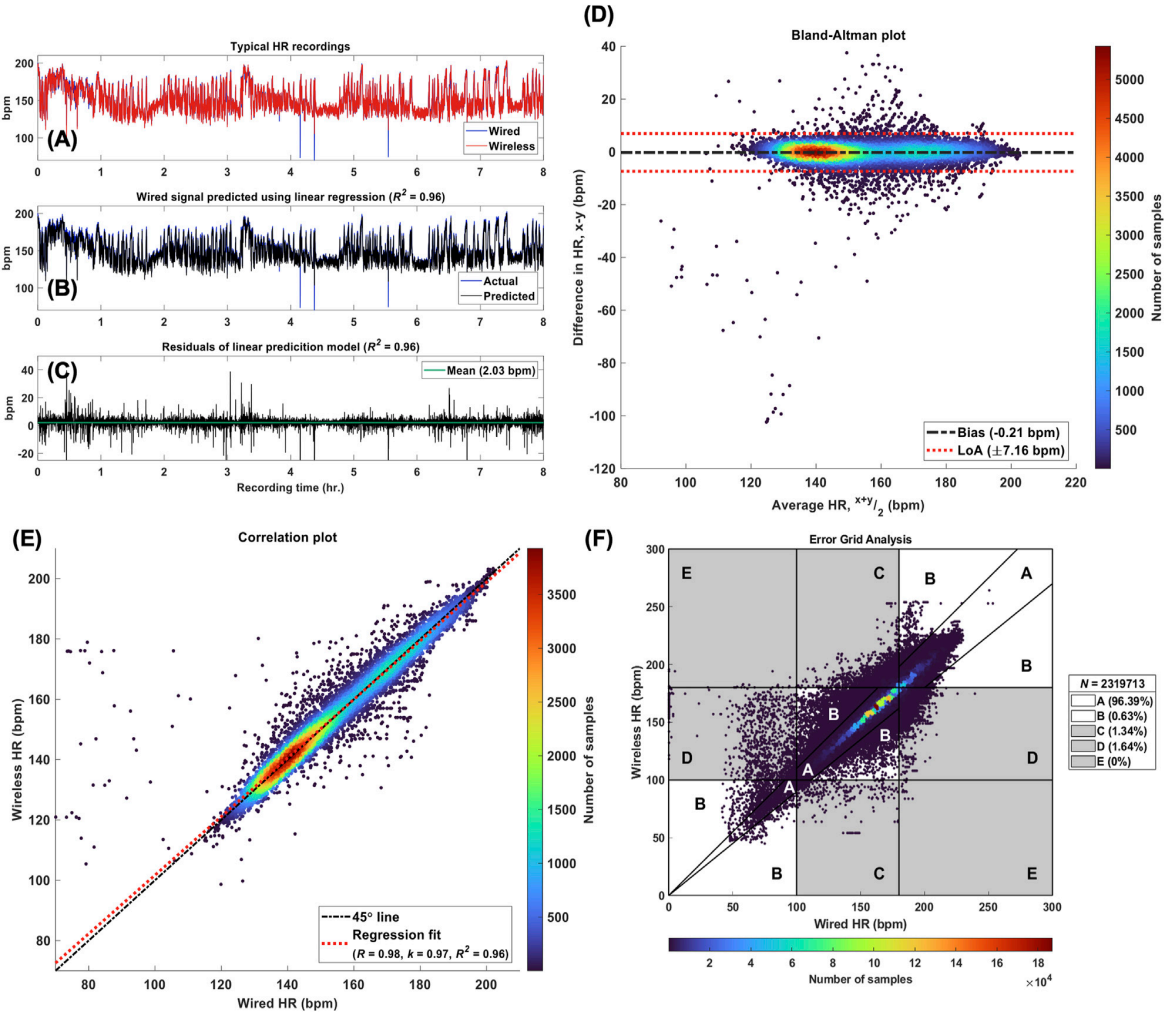
Accuracy Analysis. Legend. **(A)** Wired and wireless HR signals (x and y, respectively) from a typical patient recording. **(B)** The actual wired HR signal obtained from the patient recording and the reference signal predicted (x*) using the linear relationship: x = k. y. For this recording, k = 0.97. **(C)** The residuals of the linear model used to predict the reference signal with a mean value of 2.03 bpm and R2 = 0.96. **(D)** Bland-Altman plot for the pair of HR recordings shown in panel **(A)**, where the bias was estimated at −0.21 bpm with 95% limits of agreement (LoA) of Â±7.16 bpm. **(E)** Correlation plot for the same pair of HR signals, where the Pearson correlation coefficient was found to be 0.98. The linear regression line reflects the linear model used to predict the reference HR signal with k = 0.97. **(F)** Modified Error Grid Analysis (EGA) with the percentage of sample pairs located in regions A through E. [A, values within 10% and yielding the same clinical outcomes, B, values greater than 10% but still yielding the same clinical outcomes, C, Number of samples

Table 4 lists the median and interquartile range (IQR) for each agreement metric computed across all 96 pairs of wired and wireless HR recordings, and their respective effect sizes. Each metric demonstrated strong statistical similarity between the signals, and further validated no systematic or significant random error in the wireless recordings. The computation of these statistical measures is demonstrated in Figure 6 for a typical recording. Additionally, the Clark Error Grid revealed 97% of the data in regions A and B, i.e., HR values of wireless and corresponding wired signals yielding the same clinical outcome (Figure 6F).

**TABLE 4:** Statistical agreement metrics across all recordings and corresponding effect sizes.

Metric	Median [IQR]		Effect size
Coefficient of determination $R^{2}$	0.94	[0.90 to 0.96]	±0.03
Bias, $\bar{d}$ (bpm)	0.04	[-0.08 to 0.12]	±0.15
Upper 95%-limit of agreement (bpm)	6.36	[4.23 to 8.43]	±1.64
Lower 95-% limit of agreement (bpm)	−6.28	[-8.19 to −4.39]	

Results are presented as median [IQR]. Beats per minute (bpm).

### 5.4 Secondary outcome

We obtained a total of 23 responses from nurses and 18 from parents. Generally, both groups were very satisfied with the wireless skin sensor. Results are presented in Table 5.

**TABLE 5:** Survey responses.

	Nurse	Parent
Number of surveys completed	23	18
Satisfaction with wireless sensors (max score = 10)	9 [7.5–10]	9.5 [8–10]
Issues encountered	12 (52)	8 (44)
Adhesive	—	5 (28)
Size of sensor	7 (30)	2 (11)
Unstable signal	—	3 (17)
Sensor location	4 (17)	—

Results are presented as n (%) or median [IQR].

## 6 Discussion

The current study is a major step forward to understand the challenges associated with the development and implementation of skin wireless sensors monitoring in the NICU. For that, feasibility, safety, and accuracy were assessed over several hours and multiple days of recordings, during regular patient care in a diverse set of NICU patients. ECG and HR wireless monitoring showed very good accuracy and safety, with detection of some issues related to Bluetooth connectivity during manipulation and KC.

### 6.1 Comparison to existing research

Over the last 5 years eight studies have assessed the feasibility of six different wireless technologies in neonates (Bush et al., 2021; Henry et al., 2021; Coleman et al., 2022; Ginsburg et al., 2022b; Scholten et al., 2022; van Twist et al., 2022; Scholten et al., 2023; Thomas et al., 2024). Feasibility was assessed by either investigating the time required for device placement, time to signal acquisition, or signal coverage. The first two were mostly used in term infants in the delivery room as the ability of the sensors to quickly and reliably obtain real-time signals is important in emergency situations (Bush et al., 2021; Henry et al., 2021; van Twist et al., 2022). For routine monitoring in the NICU or nursery, overall signal coverage is more important, as signals are used throughout the day for documentation, events monitoring, and clinical decisions (Ginsburg et al., 2022b; Scholten et al., 2022; 2023; Thomas et al., 2024).

Existing studies vary significantly in their durations of recordings, which plays an important role in the interpretation of results. Some investigations had recording periods of ≤ 1 h which may have not included infant movements, different body positions, routine care, or other elements of regular NICU activity (Henry et al., 2021; Thomas et al., 2024). Two studies have included a total of 58 NICU patients [19 and 39 during regular NICU care and long recording periods (500 h–1,000 h)] (Scholten et al., 2022; 2023). The % of signal loss (lead off or BLE disconnection) was 0.8% and 2.1% for HR compared to 10.3% in our study, mostly due to BLE disconnection during KC (3% vs. 61%). However, in these two studies the antenna of the tested device was localized outside of the sensors, ensuring better wireless connectivity but making the device longer, bulkier and more difficult to handle (Scholten et al., 2022; Scholten et al., 2023). Identification of causes of signal loss are essential for improvement and further development of these devices. The major causes of HR data loss in *Sholten et al* studies were medical procedures (9%), “multiple activities” (8.7%) and nurse care (8.4%), similar to our findings of 7.3% during ‘routine care’.

Although extremely important, skin safety of novel wearable devices was rarely considered (Chung et al., 2020; Scholten et al., 2023). Furthermore, the impact of sensors removal on neonatal pain was never evaluated. Generally, studies have examined safety focused on neonatal skin condition by applying the NSCS. Chung et al used the same sensor and adhesive of our study and evaluated the NSCS in 50 NICU/PICU patients. Only two subjects (4%) exhibited a one-point increase after sensor removal compared to 47% in our study. However, in *Chung et al* study the hydrogel adhesive stayed in place for up to 24 h and the longer in contact with the skin the easier to remove. Also, pictures when taken almost 15 min after sensor removal compared to immediately in our study (Chung et al., 2020). In another study, *Sholten et al* used a dry electrode without adhesive and reported an increase of the median NSCS score from 3 [3–3] to 4 [3–4] in 5/19 (26%) of NICU patients (Scholten et al., 2022).

Accuracy was the most frequently reported outcome in studies exploring new wearable vital sign monitoring devices in neonates (Senechal et al., 2023a). In the last 5 years we identified nine studies of wireless wearable HR monitors used in neonates (Henry et al., 2021; Pike et al., 2021; Coleman et al., 2022; Ginsburg et al., 2022b; 2022a; Harrell et al., 2022; Scholten et al., 2023; Thomas et al., 2024). However, five studies utilized pulse oximeters as a reference measurement, thus limiting the ability to establish accuracy (Coleman et al., 2022; Ginsburg et al., 2022b; Ginsburg et al., 2022a; Harrell et al., 2022; Thomas et al., 2024). Overall, six different devices have been tested showing a bias ranging from −0.66 to 3.14 bpm with a 95% CI between −12.74 and 19.02 bpm compared to a median [IQR] of 0.04 bpm [-0.08–0.12] of our study (Chung et al., 2020; Henry et al., 2021; Pike et al., 2021; Coleman et al., 2022; Ginsburg et al., 2022b; 2022a; Scholten et al., 2023; Thomas et al., 2024). Studies varied significantly on their design and only five enrolled NICU/PICU patients using recording periods of 30 min to 24 h (Chung et al., 2020; Henry et al., 2021; Harrell et al., 2022; Scholten et al., 2023; Thomas et al., 2024). In three of them, the wireless device was compared to the Philips Intellivue standard of care with a bias ranging from −0.6 to 0.03 bpm and 95%CI between −5.6 and 5.0 bpm (Chung et al., 2020; Henry et al., 2021; Scholten et al., 2023). Importantly, many of these studies did not report on gestational age at birth, postmenstrual age at study, birthweight, weight at enrollment, type of respiratory support, and diagnoses at enrollment whereas our study provides detailed clinical description of the participants and included some extremely premature or unstable neonates during regular NICU care (Chung et al., 2020; Henry et al., 2021; Coleman et al., 2022; Ginsburg et al., 2022a).

Finally, some studies also examined the ability to detect important events such as tachycardias, bradycardias, and apneas. The two main methods used were: 1) event detection models which assessed the number of true positive, false positive, true negative, and false negative events and 2) Clarke Error Grids, which quantify clinical accuracy by segmenting a scatterplot of paired samples into zones describing the degree of agreement between the proposed device and the reference. We used the Clarke Error Grid due to the challenges of defining events for actual HR value thresholds, minimum duration, and discrete vs clustered events. Our results confirmed HR values of wireless and corresponding wired signals yielding the same clinical outcome.

Only one study has included assessment of healthcare provider (HCP) or parent views on the use of wireless devices in the NICU (Peterson et al., 2024). The study included NICU patients and newborns in the Delivery Room and assessed the view of 51 parents and 101 HCP. The duration of parents and HCP interaction with the device was not specified, making it difficult to assess their degree of expertise when providing feedback. Parents and HCP were generally satisfied with the wireless technology (very easy to easy) and parents reported concerns with the materials used to interface the sensor on to their baby, which was similar to the expressed by our parents with the adhesive.

### 6.2 Study limitations

The current study had some limitations. It was challenging to recruit more acute patients such as extremely premature infants on various forms of invasive ventilation thus limiting the generalizability of these findings for these high-risk patients. Multiple researchers completed the NIPS at the end of each day of recording, which introduces variability due to raters’ interpretations. Another limitation is that the skin safety metrics were not applied to the 3-lead electrodes used in the standard of care limiting the ability to compare with the standard of care. Importantly, this prospective study provides a detailed and comprehensive evaluation of the use of a new wireless skin sensor for ECG and HR monitoring, with long recordings done during routine care in a real-life NICU environment and a variety of neonates receiving different types of treatment. Also, a customized data acquisition system allowed for simultaneous recordings of HR and ECG signals from the wired and wireless and re-sample was done making possible a sample-to-sample comparison of the devices avoiding indirect methods such as video recording of patient monitors, manual synchronization, or signal averaging schemes to compare signals (Henry et al., 2021; Pike et al., 2021; Harrell et al., 2022). Additionally, differently from other studies (Chung et al., 2020; Coleman et al., 2022; Ginsburg et al., 2022a; Harrell et al., 2022; Thomas et al., 2024), all recoded data was analyzed and reported on results, including segments of infants’ movements and handling, therefore expanding the applicability of the results.

### 6.3 Future directions

Bluetooth disconnections during KC and nursing interventions were mostly less than 5 s but occurred in a large percentage of time. In more unstable patients this could affect the precision in timely detect any ECG/HR abnormality that requires medical intervention. In more stable infants such as in the current study, it is unlikely that these gaps would have such impact. Nevertheless, we believe that this issue should be solved. Maybe this can be done by changing the location of the antenna in the sensor or by using a more potent antenna. Furthermore, the potential for integrating machine learning algorithms to improve signal processing and reduce gaps in coverage should be explored. Moreover, sensors should be tested in unstable and more premature patients and include monitoring of additional signals.

## 7 Conclusion

In conclusion, ECG and HR monitoring of NICU patients using a new wireless skin sensor was feasible, safe, and accurate when compared to the wired standard signals. The current findings are a major step forward to understand the challenges associated with the development and implementation of skin wireless sensors monitoring in the NICU. The use of this technology may help decrease complications associated with the wires and cables such as tangling and pain, and parents’ anxiety and fear with the NICU technology. Limitations include challenges in recruiting unstable and very immature neonates, subjectivity of pain assessments, and inability to apply safety metrics to the wired system. Future adjustments in the technology are needed to improve signal coverage during KC, test the sensors in unstable and more premature patient, and include monitoring of additional signs.

## Data Availability

The raw data supporting the conclusions of this article will be made available upon reasonable request.
