# Supplementary material for: Wireless skin sensors for electrocardiogram and heart rate monitoring in the neonatal intensive care unit: a prospective feasibility, safety, and accuracy study
Source: Front Bioeng Biotechnol. 2025 Apr 29;13:1555882. doi: 10.3389/fbioe.2025.1555882 (PMC12069355; doi:10.3389/fbioe.2025.1555882)
Supplement: Supplementary file 1 [file Table1.docx]

**DATA COLLECTION FORM (PHASE 1)**

**BASIC STUDY INFORMATION**

Patient ID: _____________

Date and time of study initiation: _____________ (YYYY-MM-DD) ____________ (HH:MM)

Date and time of study completion: _____________ (YYYY-MM-DD) ____________ (HH:MM)

**DEMOGRAPHICS**

| Year of birth: _______ | Sex: ☐ Male ☐ Female | Hours of life (at enrollment): ________ |
| --- | --- | --- |
| Gestational age (at birth): ______ weeks ______ days | | Birth weight: _________ grams |
| Gestational age (corrected): ______ weeks ______ days | |  |
| Diagnosis(es) at time of enrollment: | | |
| ☐ Perinatal asphyxia ☐ Anemia ☐ Apneas and bradycardias ☐ Bronchopulmonary dysplasia | | |
| ☐ Hydrocephalus ☐ Intraventricular hemorrhage ☐ Jaundice ☐ Necrotizing enterocolitis | | |
| ☐ Other, specify: ______________________________ | | |
| Treatments at enrollment: | | |
| ☐ Therapeutic hypothermia | | ☐ Continuous positive airway pressure (CPAP) |
| ☐ Conventional mechanical ventilation (CMV) | | ☐ High frequency ventilation (HFV) |
| ☐ Nasal intermittent positive pressure ventilation (NIPPV) | | ☐ Not applicable/healthy infants |
| Patient group: ☐ A ☐ B ☐ C ☐ D ☐ E ☐ F ☐ G ☐ H | | |

| A = healthy term infants in room air at enrollment  B = term infants with perinatal asphyxia undergoing therapeutic hypothermia at enrollment  C = healthy preterm infants in room air at enrollment  D = preterm infants on continuous positive airway pressure at enrollment  E = extremely preterm infants on conventional mechanical ventilation at enrollment  F = extremely preterm infants on high frequency ventilation at enrollment  G = extremely preterm infants on nasal intermittent positive end expiratory pressure at enrollment  H = extremely preterm infants on continuous positive airway pressure at enrollment |
| --- |

**HOURLY LOGS**

Day 1:

| Date: Start Time: End Time: | | | | | | | | | |
| --- | --- | --- | --- | --- | --- | --- | --- | --- | --- |
|  | baseline | 1h | 2h | 3h | 4h | 5h | 6h | 7h | 8h |
| Location of chest unit  (Center, R or L side) |  |  |  |  |  |  |  |  |  |
| Location of limb unit (R-right, L-left, H-hand/wrist, F-foot) |  |  |  |  |  |  |  |  |  |
| Number of conventional wires |  |  |  |  |  |  |  |  |  |
| Axillary temperature (°C) |  |  |  |  |  |  |  |  |  |
| Room temperature (°C) |  |  |  |  |  |  |  |  |  |
| Air temperature (°C) & humidity (%) - 15cm from neonate |  |  |  |  |  |  |  |  |  |
| Kangaroo care  (Y/N) |  |  |  |  |  |  |  |  |  |
| Nursing care  (Y/N) |  |  |  |  |  |  |  |  |  |
| Medications  (Write down any medication in use) |  |  |  |  |  |  |  |  |  |
| Phototherapy  (Y/N) |  |  |  |  |  |  |  |  |  |
| Type of ventilatory support (CPAP, CMV, HFV, NIPPV) |  |  |  |  |  |  |  |  |  |
| Neonate location  (incubator/crib) |  |  |  |  |  |  |  |  |  |
| Neonate position  (s-supine, p-prone, side) |  |  |  |  |  |  |  |  |  |
| Sensor disconnection (N)/reasons* |  |  |  |  |  |  |  |  |  |

*A = x-ray, B = bath, C = cleaning, D = tests, E = transport, F = other

Photographs of the skin at sensor placement sites (place “✓” if done)

| Chest | ☐ Baseline (0h) | Limb | ☐ Baseline (0h) |
| --- | --- | --- | --- |
|  | ☐ 8h |  | ☐ 8h |

Neonatal Infant Pain Scale – Day 1

| **Pain Assessment** | | Score | |
| --- | --- | --- | --- |
| **Facial Expression** | |  | |
| 0 – Relaxed muscles | Restful face, neutral expression | | |
| 1 – Grimace | Tight facial muscles, furrowed brow, chin, jaw, (negative facial expression – nose, mouth, brow) | | |
| **Cry** | |  | |
| 0 – No cry | Quiet, not crying | | |
| 1 - Whimper | Mild moaning, intermittent | | |
| 2 – Vigorous Cry | Loud scream; rising, shrill, continuous (Note: silent cry may be scored if baby is intubated as evidenced by obvious mouth and facial movement.) | | |
| **Breathing Patterns** | |  | |
| 0 – Relaxed | Usual pattern for this infant | | |
| 1 – Change in Breathing | Indrawing, irregular, faster than usual, gagging, breath holding | | |
| **Arms** | |  | |
| 0 – Relaxed/Restrained | No muscular rigidity, occasional random movements of arms | | |
| 1 – Flexed/Extended | Tense, straight legs; rigid and/or rapid extension/flexion | | |
| **Legs** | |  | |
| 0 – Relaxed/Restrained | No muscular rigidity, occasional random movements of legs | | |
| 1 – Flexed/Extended | Tense, straight legs; rigid and/or rapid extension/flexion | | |
| **State of Arousal** | |  | |
| 0 – Sleeping/Awake | Quiet, peaceful, sleeping, or alert random leg movement | | |
| 1 – Fussy | Alert, restless, thrashing | | |
| **TOTAL** | | |  |

Rater: ____________

Day 2:

| Date: Start Time: End Time: | | | | | | | | | |
| --- | --- | --- | --- | --- | --- | --- | --- | --- | --- |
|  | baseline | 1h | 2h | 3h | 4h | 5h | 6h | 7h | 8h |
| Location of chest unit  (Center, R or L side) |  |  |  |  |  |  |  |  |  |
| Location of limb unit (R-right, L-left, H-hand/wrist, F-foot) |  |  |  |  |  |  |  |  |  |
| Number of conventional wires |  |  |  |  |  |  |  |  |  |
| Axillary temperature (°C) |  |  |  |  |  |  |  |  |  |
| Room temperature (°C) |  |  |  |  |  |  |  |  |  |
| Air temperature (°C) & humidity (%) - 15cm from neonate |  |  |  |  |  |  |  |  |  |
| Kangaroo care  (Y/N) |  |  |  |  |  |  |  |  |  |
| Nursing care  (Y/N) |  |  |  |  |  |  |  |  |  |
| Medications  (Write down any medication in use) |  |  |  |  |  |  |  |  |  |
| Phototherapy  (Y/N) |  |  |  |  |  |  |  |  |  |
| Type of ventilatory support (CPAP, CMV, HFV, NIPPV) |  |  |  |  |  |  |  |  |  |
| Neonate location  (incubator/crib) |  |  |  |  |  |  |  |  |  |
| Neonate position  (s-supine, p-prone, side) |  |  |  |  |  |  |  |  |  |
| Sensor disconnection (N)/reasons* |  |  |  |  |  |  |  |  |  |

*A = x-ray, B = bath, C = cleaning, D = tests, E = transport, F = other

Photographs of the skin at sensor placement sites (place “✓” if done)

| Chest | ☐ Baseline (0h) | Limb | ☐ Baseline (0h) |
| --- | --- | --- | --- |
|  | ☐ 8h |  | ☐ 8h |

Neonatal Infant Pain Scale – Day 2

| **Pain Assessment** | | Score | |
| --- | --- | --- | --- |
| **Facial Expression** | |  | |
| 0 – Relaxed muscles | Restful face, neutral expression | | |
| 1 – Grimace | Tight facial muscles, furrowed brow, chin, jaw, (negative facial expression – nose, mouth, brow) | | |
| **Cry** | |  | |
| 0 – No cry | Quiet, not crying | | |
| 1 - Whimper | Mild moaning, intermittent | | |
| 2 – Vigorous Cry | Loud scream; rising, shrill, continuous (Note: silent cry may be scored if baby is intubated as evidenced by obvious mouth and facial movement.) | | |
| **Breathing Patterns** | |  | |
| 0 – Relaxed | Usual pattern for this infant | | |
| 1 – Change in Breathing | Indrawing, irregular, faster than usual, gagging, breath holding | | |
| **Arms** | |  | |
| 0 – Relaxed/Restrained | No muscular rigidity, occasional random movements of arms | | |
| 1 – Flexed/Extended | Tense, straight legs; rigid and/or rapid extension/flexion | | |
| **Legs** | |  | |
| 0 – Relaxed/Restrained | No muscular rigidity, occasional random movements of legs | | |
| 1 – Flexed/Extended | Tense, straight legs; rigid and/or rapid extension/flexion | | |
| **State of Arousal** | |  | |
| 0 – Sleeping/Awake | Quiet, peaceful, sleeping, or alert random leg movement | | |
| 1 – Fussy | Alert, restless, thrashing | | |
| **TOTAL** | | |  |

Rater: ____________

Day 3:

| Date: Start Time: End Time: | | | | | | | | | |
| --- | --- | --- | --- | --- | --- | --- | --- | --- | --- |
|  | baseline | 1h | 2h | 3h | 4h | 5h | 6h | 7h | 8h |
| Location of chest unit  (Center, R or L side) |  |  |  |  |  |  |  |  |  |
| Location of limb unit (R-right, L-left, H-hand/wrist, F-foot) |  |  |  |  |  |  |  |  |  |
| Number of conventional wires |  |  |  |  |  |  |  |  |  |
| Axillary temperature (°C) |  |  |  |  |  |  |  |  |  |
| Room temperature (°C) |  |  |  |  |  |  |  |  |  |
| Air temperature (°C) & humidity (%) - 15cm from neonate |  |  |  |  |  |  |  |  |  |
| Kangaroo care  (Y/N) |  |  |  |  |  |  |  |  |  |
| Nursing care  (Y/N) |  |  |  |  |  |  |  |  |  |
| Medications  (Write down any medication in use) |  |  |  |  |  |  |  |  |  |
| Phototherapy  (Y/N) |  |  |  |  |  |  |  |  |  |
| Type of ventilatory support (CPAP, CMV, HFV, NIPPV) |  |  |  |  |  |  |  |  |  |
| Neonate location  (incubator/crib) |  |  |  |  |  |  |  |  |  |
| Neonate position  (s-supine, p-prone, side) |  |  |  |  |  |  |  |  |  |
| Sensor disconnection (N)/reasons* |  |  |  |  |  |  |  |  |  |

*A = x-ray, B = bath, C = cleaning, D = tests, E = transport, F = other

Photographs of the skin at sensor placement sites (place “✓” if done)

| Chest | ☐ Baseline (0h) | Limb | ☐ Baseline (0h) |
| --- | --- | --- | --- |
|  | ☐ 8h |  | ☐ 8h |

Neonatal Infant Pain Scale – Day 3

| **Pain Assessment** | | Score | |
| --- | --- | --- | --- |
| **Facial Expression** | |  | |
| 0 – Relaxed muscles | Restful face, neutral expression | | |
| 1 – Grimace | Tight facial muscles, furrowed brow, chin, jaw, (negative facial expression – nose, mouth, brow) | | |
| **Cry** | |  | |
| 0 – No cry | Quiet, not crying | | |
| 1 - Whimper | Mild moaning, intermittent | | |
| 2 – Vigorous Cry | Loud scream; rising, shrill, continuous (Note: silent cry may be scored if baby is intubated as evidenced by obvious mouth and facial movement.) | | |
| **Breathing Patterns** | |  | |
| 0 – Relaxed | Usual pattern for this infant | | |
| 1 – Change in Breathing | Indrawing, irregular, faster than usual, gagging, breath holding | | |
| **Arms** | |  | |
| 0 – Relaxed/Restrained | No muscular rigidity, occasional random movements of arms | | |
| 1 – Flexed/Extended | Tense, straight legs; rigid and/or rapid extension/flexion | | |
| **Legs** | |  | |
| 0 – Relaxed/Restrained | No muscular rigidity, occasional random movements of legs | | |
| 1 – Flexed/Extended | Tense, straight legs; rigid and/or rapid extension/flexion | | |
| **State of Arousal** | |  | |
| 0 – Sleeping/Awake | Quiet, peaceful, sleeping, or alert random leg movement | | |
| 1 – Fussy | Alert, restless, thrashing | | |
| **TOTAL** | | |  |

Rater: ____________

Day 4:

| Date: Start Time: End Time: | | | | | | | | | |
| --- | --- | --- | --- | --- | --- | --- | --- | --- | --- |
|  | baseline | 1h | 2h | 3h | 4h | 5h | 6h | 7h | 8h |
| Location of chest unit  (Center, R or L side) |  |  |  |  |  |  |  |  |  |
| Location of limb unit (R-right, L-left, H-hand/wrist, F-foot) |  |  |  |  |  |  |  |  |  |
| Number of conventional wires |  |  |  |  |  |  |  |  |  |
| Axillary temperature (°C) |  |  |  |  |  |  |  |  |  |
| Room temperature (°C) |  |  |  |  |  |  |  |  |  |
| Air temperature (°C) & humidity (%) - 15cm from neonate |  |  |  |  |  |  |  |  |  |
| Kangaroo care  (Y/N) |  |  |  |  |  |  |  |  |  |
| Nursing care  (Y/N) |  |  |  |  |  |  |  |  |  |
| Medications  (Write down any medication in use) |  |  |  |  |  |  |  |  |  |
| Phototherapy  (Y/N) |  |  |  |  |  |  |  |  |  |
| Type of ventilatory support (CPAP, CMV, HFV, NIPPV) |  |  |  |  |  |  |  |  |  |
| Neonate location  (incubator/crib) |  |  |  |  |  |  |  |  |  |
| Neonate position  (s-supine, p-prone, side) |  |  |  |  |  |  |  |  |  |
| Sensor disconnection (N)/reasons* |  |  |  |  |  |  |  |  |  |

*A = x-ray, B = bath, C = cleaning, D = tests, E = transport, F = other

Photographs of the skin at sensor placement sites (place “✓” if done)

| Chest | ☐ Baseline (0h) | Limb | ☐ Baseline (0h) |
| --- | --- | --- | --- |
|  | ☐ 8h |  | ☐ 8h |

Neonatal Infant Pain Scale – Day 4

| **Pain Assessment** | | Score | |
| --- | --- | --- | --- |
| **Facial Expression** | |  | |
| 0 – Relaxed muscles | Restful face, neutral expression | | |
| 1 – Grimace | Tight facial muscles, furrowed brow, chin, jaw, (negative facial expression – nose, mouth, brow) | | |
| **Cry** | |  | |
| 0 – No cry | Quiet, not crying | | |
| 1 - Whimper | Mild moaning, intermittent | | |
| 2 – Vigorous Cry | Loud scream; rising, shrill, continuous (Note: silent cry may be scored if baby is intubated as evidenced by obvious mouth and facial movement.) | | |
| **Breathing Patterns** | |  | |
| 0 – Relaxed | Usual pattern for this infant | | |
| 1 – Change in Breathing | Indrawing, irregular, faster than usual, gagging, breath holding | | |
| **Arms** | |  | |
| 0 – Relaxed/Restrained | No muscular rigidity, occasional random movements of arms | | |
| 1 – Flexed/Extended | Tense, straight legs; rigid and/or rapid extension/flexion | | |
| **Legs** | |  | |
| 0 – Relaxed/Restrained | No muscular rigidity, occasional random movements of legs | | |
| 1 – Flexed/Extended | Tense, straight legs; rigid and/or rapid extension/flexion | | |
| **State of Arousal** | |  | |
| 0 – Sleeping/Awake | Quiet, peaceful, sleeping, or alert random leg movement | | |
| 1 – Fussy | Alert, restless, thrashing | | |
| **TOTAL** | | |  |

Rater: ____________**ADVERSE EVENTS**

☐ Skin irritation/redness

If observed, complete section below:

| Onset date: ________________ (YYYY-MM-DD) | Location: ________________ |
| --- | --- |
| Severity: ☐ Mild ☐ Moderate ☐ Severe | Treatment required? ☐ Yes ☐ No |
| Relationship to study sensors: ☐ Related ☐ Suspected ☐ Not suspected | |
| Outcome: ☐ Recovered ☐ Improving ☐ Unchanged ☐ End of study participation | |

☐ Skin erosions

If observed, complete section below:

| Onset date: ________________ (YYYY-MM-DD) | Location: ________________ |
| --- | --- |
| Severity: ☐ Mild ☐ Moderate ☐ Severe | Treatment required? ☐ Yes ☐ No |
| Relationship to study sensors: ☐ Related ☐ Suspected ☐ Not suspected | |
| Outcome: ☐ Recovered ☐ Improving ☐ Unchanged ☐ End of study participation | |

☐ Skin bleeding

If observed, complete section below:

| Onset date: ________________ (YYYY-MM-DD) | Location: ________________ |
| --- | --- |
| Severity: ☐ Mild ☐ Moderate ☐ Severe | Treatment required? ☐ Yes ☐ No |
| Relationship to study sensors: ☐ Related ☐ Suspected ☐ Not suspected | |
| Outcome: ☐ Recovered ☐ Improving ☐ Unchanged ☐ End of study participation | |

☐ Other, specify: ___________________
