# Supplementary material for: Wireless skin sensors for electrocardiogram and heart rate monitoring in the neonatal intensive care unit: a prospective feasibility, safety, and accuracy study
Source: Front Bioeng Biotechnol. 2025 Apr 29;13:1555882. doi: 10.3389/fbioe.2025.1555882 (PMC12069355; doi:10.3389/fbioe.2025.1555882)
Supplement: Supplementary file 2 [file Table2.docx]

| **CODE** | **Reason** |
| --- | --- |
| AF | Biodash Application Failure/Malfunction |
| CP-ECG | Change of ECG probes |
| CP-RR | Change in respiratory probes |
| CP-SpO2 | Change of SpO2 probe |
| CP-T | Change of temperature probe |
| PS | Poor Sensor Signal |
| SR-adj | Sensor readjustment |
| SR-rep | Sensor replacement |
| -SX | Sensor removal |
| B | Bathing |
| BT | Blood Test (via heel stick or other local acute extraction method) |
| C | Crying – inconsistent |
| DC | Diaper change |
| KC | Kangaroo care |
| PP | Prone position |
| SP | Supine position |
| SS | Side position |
| RC | Routine Care |
| S | Sleeping |
| BF | Breastfeeding |
| FB | Feeding by bottle |
| G | Gavage |
| AC | Airway care |
| U | Ultrasound |
| XR | X-Ray |
| EXAM | 10 min |
| PT | Phototherapy |
| I | Intubation |
| E | Extubation |
| CS | Clinical seizure |
| O | Other |
