# Supplementary material for: Wireless skin sensors for electrocardiogram and heart rate monitoring in the neonatal intensive care unit: a prospective feasibility, safety, and accuracy study
Source: Front Bioeng Biotechnol. 2025 Apr 29;13:1555882. doi: 10.3389/fbioe.2025.1555882 (PMC12069355; doi:10.3389/fbioe.2025.1555882)
Supplement: Supplementary file 3 [file Table5.docx]

**Fig 1** Boxplot of skin scores per day of recording

**Fig 2** Boxplot of Neonatal Infant Pain Score (NIPS per rater)

**Fig 3** Boxplot of Neonatal Infant Pain Score (NIPS) per day of recording

**Fig 4** Heatmap of NSCS and NIPS scores
