# Supplementary material for: Wireless skin sensors for electrocardiogram and heart rate monitoring in the neonatal intensive care unit: a prospective feasibility, safety, and accuracy study
Source: Front Bioeng Biotechnol. 2025 Apr 29;13:1555882. doi: 10.3389/fbioe.2025.1555882 (PMC12069355; doi:10.3389/fbioe.2025.1555882)
Supplement: Supplementary file 4 [file Table3.docx]

Clarke Error Grid modifications

| **Region** | | **Modifications** | **Original** | |
| --- | --- | --- | --- | --- |
| **A** | Values within 10% of the reference device and yielding the same clinical outcomes | | | Values within 10% of the reference device |
| **B** | Values greater than 10% of the reference device but still yielding the same clinical outcomes | | | Values greater than 20% of the reference device but still yielding the same clinical outcomes |
| **C** | Values that would result in unnecessary treatment (i.e., false positives) | | | Values that would result in unnecessary treatment (i.e., false positives) |
| **D** | Values that would result in failure-to-treat (i.e., false negatives) | | | Values that would result in failure-to-treat (i.e., false negatives) |
| **E** | Values that would result in the reverse treatment | | | Values that would result in the reverse treatment |
