# Supplementary material for: Wireless skin sensors for electrocardiogram and heart rate monitoring in the neonatal intensive care unit: a prospective feasibility, safety, and accuracy study
Source: Front Bioeng Biotechnol. 2025 Apr 29;13:1555882. doi: 10.3389/fbioe.2025.1555882 (PMC12069355; doi:10.3389/fbioe.2025.1555882)
Supplement: Supplementary file 5 [file Table4.docx]

**Supplementary 1 -** Parent Survey

**Wireless NICU Project – Survey**

Dear Parent (or legal guardian),

Thank you again for agreeing to have your baby take part in this research project. Now that your baby’s participation is complete, we are surveying your opinions of the wireless sensors we used in this project for your baby’s vital sign monitoring. This is an anonymous survey and will take you approximately 1-2 minutes to complete. Your responses will be kept confidential and with the principal investigators at the McGill University Health Center for a period of 7 years.

Sincerely,

**Principal Investigators**: Guilherme Sant’Anna (MD, PhD); Wissam Shalish (MD, PhD)

Q1. Were you satisfied with the wireless sensors placed on your baby during his/her participation in this project? Please circle the appropriate number below.

| 0 | 1 | 2 | 3 | 4 | 5 | 6 | 7 | 8 | 9 | 10 |
| --- | --- | --- | --- | --- | --- | --- | --- | --- | --- | --- |

Not satisfied at all Neutral Very satisfied

Q2. What are the things you liked about the wireless sensors?

_________________________________________________________________________________

_________________________________________________________________________________

Q3. Did you encounter any problems with the wireless sensors?

☐ Yes ☐ No

If you responded *Yes* to the question above, please specify:

_________________________________________________________________________________

_________________________________________________________________________________

**Supplementary 1 –** Nurse Survey

**Wireless NICU Project – Survey**

Dear Colleague,

We are surveying your opinions of the wireless monitoring devices we used in the Wireless NICU project. This research will serve as a first step towards exploring the feasibility of using wireless sensors on neonates for continuous vital sign monitoring. This is an anonymous survey and will take you approximately 1-2 minutes to complete. Your responses will be kept confidential and with the principal investigators at the McGill University Health Center for a period of 7 years.

Your consent for your responses to be included as part of the study results is inferred through completion of this survey.

Sincerely,

**Principal Investigators**: Guilherme Sant’Anna (MD, PhD); Wissam Shalish (MD, PhD)

Q1. Were you satisfied with the wireless sensors placed on neonates participated in this project? Please circle the appropriate number below.

| 0 | 1 | 2 | 3 | 4 | 5 | 6 | 7 | 8 | 9 | 10 |
| --- | --- | --- | --- | --- | --- | --- | --- | --- | --- | --- |

Not satisfied at all Neutral Very satisfied

Q2. What are the things you liked about the wireless sensors?

_________________________________________________________________________________

_________________________________________________________________________________

Q3. Did you encounter any problems with the wireless sensors?

☐ Yes ☐ No

If you responded *Yes* to the question above, please specify:

_________________________________________________________________________________

_________________________________________________________________________________
